# Systematic Review and Meta-Analysis of Outcomes after Cardiopulmonary Arrest in Childhood

**DOI:** 10.1371/journal.pone.0130327

**Published:** 2015-06-24

**Authors:** Robert S. Phillips, Bryonnie Scott, Simon J. Carter, Matthew Taylor, Eleanor Peirce, Patrick Davies, Ian K. Maconochie

**Affiliations:** 1 Centre for Reviews and Dissemination, University of York, York, United Kingdom; 2 Leeds Children’s Hospital, Leeds General Infirmary, Leeds, United Kingdom; 3 York District General Hospital, York, United Kingdom; 4 Hull Royal Infirmary, Kingston-upon-Hull, United Kingdom; 5 Sheffield Children’s Hospital, Sheffield, United Kingdom; 6 Nottingham Children's Hospital, Nottingham Hospitals NHS Trust, Nottingham, United Kingdom; 7 St Mary’s Hospital, Imperial College NHS Healthcare Trust, London, United Kingdom; Sapienza University of Rome, ITALY

## Abstract

**Background:**

Cardiopulmonary arrest in children is an uncommon event, and often fatal. Resuscitation is often attempted, but at what point, and under what circumstances do continued attempts to re-establish circulation become futile? The uncertainty around these questions can lead to unintended distress to the family and to the resuscitation team.

**Objectives:**

To define the likely outcomes of cardiopulmonary resuscitation in children, within different patient groups, related to clinical features.

**Data Sources:**

MEDLINE, MEDLINE in-Process & Other non-Indexed Citations, EMBASE, Cochrane database of systematic reviews and Cochrane central register of trials, Database of Abstracts of Reviews of Effects (DARE), the Health Technology Assessment database, along with reference lists of relevant systematic reviews and included articles.

**Study Eligibility Criteria:**

Prospective cohort studies which derive or validate a clinical prediction model of outcome following cardiopulmonary arrest.

**Participants and Interventions:**

Children or young people (aged 0 – 18 years) who had cardiopulmonary arrest and received an attempt at resuscitation, excluding resuscitation at birth.

**Study Appraisal and Synthesis Methods:**

Risk of bias assessment developed the Hayden system for non-randomised studies and QUADAS2 for decision rules. Synthesis undertaken by narrative, and random effects meta-analysis with the DerSimonian-Laird estimator.

**Results:**

More than 18,000 episodes in 16 data sets were reported. Meta-analysis was possible for survival and one neurological outcome; others were reported too inconsistently. In-hospital patients (average survival 37.2% (95% CI 23.7 to 53.0%)) have a better chance of survival following cardiopulmonary arrest than out-of-hospital arrests (5.8% (95% CI 3.9% to 8.6%)). Better neurological outcome was also seen, but data were too scarce for meta-analysis (17% to 71% ‘good’ outcomes, compared with 2.8% to 3.2%).

**Limitation:**

Lack of consistent outcome reporting and short-term neurological outcome measures limited the strength of conclusions that can be drawn from this review.

**Conclusions and Implications of Key Findings:**

There is a need to collaboratively, prospectively, collect potentially predictive data on these rare events to understand more clearly the predictors of survival and long-term neurological outcome.

**Systematic Review Registration Number:**

PROSPERO 2013:CRD42013005102

## Introduction

Cardiopulmonary arrest in children is an uncommon event [1–3]. When it does happen, algorithms provide structured approaches to reverse the situation [4–8], but at what point, and under what circumstances do continued attempts to re-establish circulation become futile? What are the predictive factors for a satisfactory outcome? What should those outcomes be? The current uncertainty around these questions results in discussion, and sometimes confusion during attempted resuscitations. This can lead to unintended distress to the family and to the resuscitation team.

To reduce this uncertainty and to address these questions, the literature on potential predictive factors such as the patient’s characteristics, the nature of cardiopulmonary arrest, and the outcomes (not only survival but the neurological status and quality of life for the patient sometime after the event) may be considered, as they may assist decision making about continued resuscitation.

Previous studies have concluded with varying estimates of the nature of outcomes following cardiopulmonary arrest[3, 9–11], with grossly different outcomes from in-hospital[11] versus out-of-hospital[10] arrest and uncertainty about the effect of bystander resuscitation[10] amongst other factors. Some of these uncertainties may be lessened by a systematic review to collect, appraise and synthesise the data and subsequently be used to inform healthcare workers (and parents/carers) in delivering care in an evidence based manner.

This systematic review therefore is needed to review what the current evidence is and if it can assist in key clinical decisions that occur in managing a paediatric patient with cardiopulmonary resuscitation.

## Methods

This review followed “Systematic reviews: CRD's guidance for undertaking reviews in health care”[12] and was registered prospectively on PROSPERO (CRD: 42013005102). It sought prospective cohort studies which examined predictor variables, or derived or validated a clinical prediction model, for outcomes following cardiopulmonary arrest in children and young people in whom there was an attempt at resuscitation. ‘Attempt at resuscitation’ was defined as all cardiopulmonary resuscitation protocols, including the use of positive pressure ventilation, cardiac compressions and resuscitation drugs. Studies with adults which reported data for patients under 18 years old were included if outcome data were reported separately for children. Resuscitation at birth was excluded. The principal outcomes examined were: return of spontaneous circulation (ROSC); survival at 30 days, to hospital discharge, at one year and at five years or other duration post event/discharge; duration of admission; neurological and functional outcome at 30 days, at one year, five years or other duration; and, quality of life measurements.

We aimed to research after the dissemination of ‘Guidelines for cardiopulmonary resuscitation and emergency cardiovascular care: an international consensus on science’ [4, 5] and so limited our review to studies published after 2003.

### Search and identification strategy

We searched Ovid MEDLINE, MEDLINE in-process and other non-indexed citations, EMBASE, EMBASE Classic, Cochrane Database of Systematic Reviews (2005 to June 2013), CENTRAL (via the Cochrane Library), Evidence Based Medicine Reviews, including ACP Journal Club, Database of Abstracts of Reviews of Effects (DARE), the Health Technology Assessment database. The search was updated on the Medline database in November 2014. See Appendix 1 for an example search strategy. In addition, reference lists of relevant systematic reviews and included articles were also reviewed. Both published and unpublished studies were sought and no language restrictions were applied. Non-English papers were translated where necessary.

Two reviewers independently screened the titles and abstracts of studies for inclusion. Disagreements were resolved by consensus, or when this proved impossible, were to be resolved by recourse to an independent adjudicator.

### Methodological quality assessment and data extraction

Data were extracted by one researcher using a standardised data extraction form and checked by a second. Data extracted included patient demographics, clinical characteristics and details of study conduct. In addition, information was extracted on; the methods used to measure the predictor variables and the outcome assessments; the nature and duration of follow-up; missing data and patients lost to follow-up; how any subgroups were chosen; methods of statistical analysis, including variables adjusted for, and the number of patients included.

Risk of bias assessment was undertaken using a modified Hayden system for non-randomised studies [13]. The protocol made allowance for examining clinical prediction rules using QUADAS2 [14].

### Method of analysis/synthesis

Where appropriate data were available, meta-analysis of proportions was undertaken with random-effect methods of the natural log-odds (logit) of the proportion and odds ratios with the DerSimonian-Laird estimator, assuming a normal distribution of the log (odds) [12] using the ‘R’ statistical environment. Other methods, for example, assessing the diagnostic accuracy of any derived clinical decision rules, had been previously specified. Planned subgroup analysis included age, co-morbidity, location of resuscitation and nature of cardiopulmonary arrest was undertaken. Publication bias was not quantitatively assessed through insufficient data.

Heterogeneity was explored through consideration of study populations (both the geographical location and nature of the diseases under study), study design, predictor variables assessed and outcomes chosen.

For those areas where a quantitative synthesis was not possible, a narrative approach was used.

## Results

**Fig 1:**
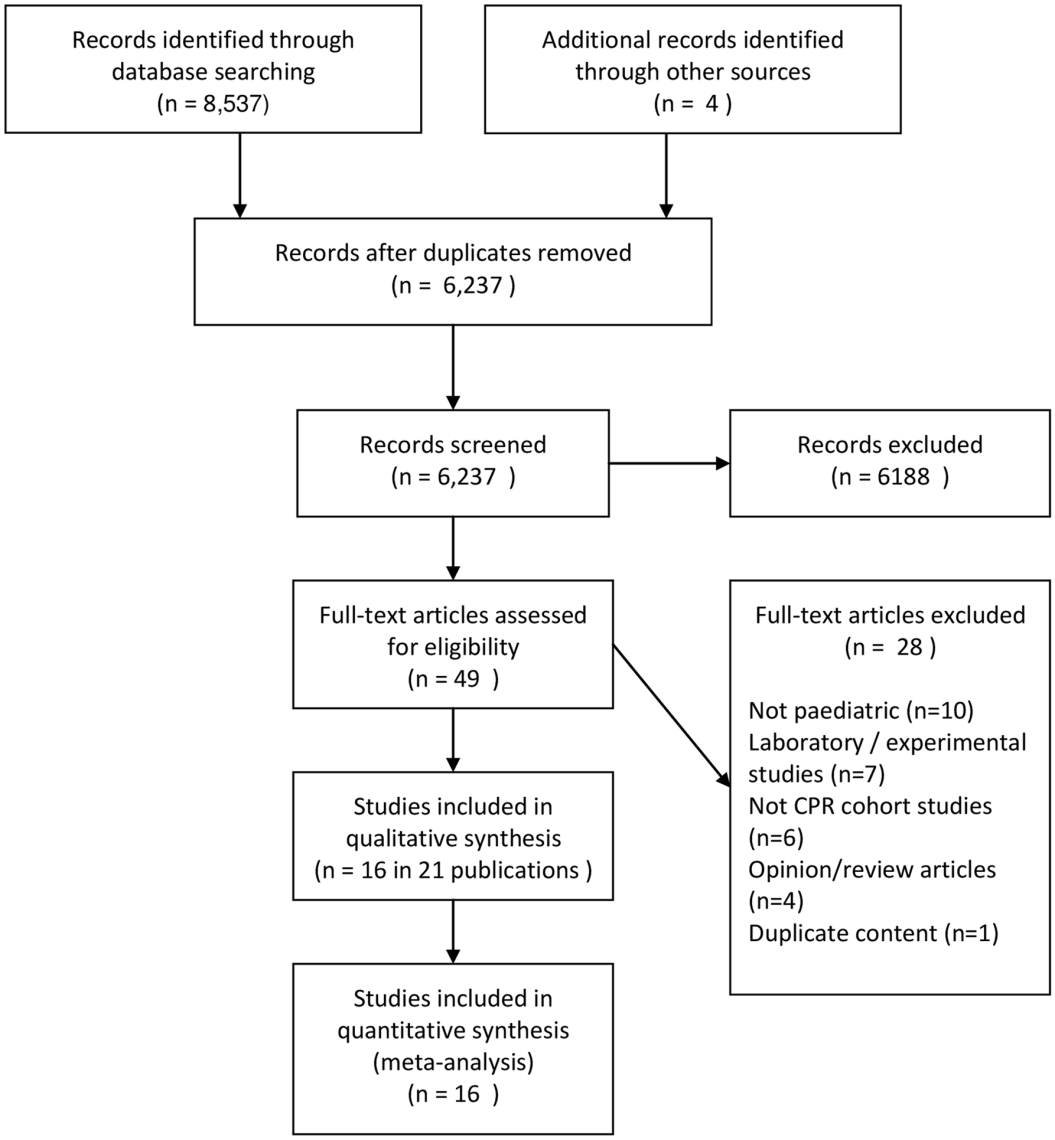
Flow of identified articles.

6233 articles were identified with this search, of which 21 papers were eventually included[1–3, 9–11, 15–29] reporting on 16 data sets (see Fig 1).

The studies included over 18,000 episodes of cardiopulmonary arrest, and provided data on out of hospital arrests, arrests throughout hospitals, paediatric intensive care arrests, and specific situations (arrests due to trauma, or only witnessed arrests). The outcomes reported included mortality and neurological status, at a range of time periods including discharge, one month, six months and one year. The studies reported on between 26 and 5158 (median 250) arrests.

The assessment of risk of bias found case selection to be appropriate, and the outcome measurement of survival and major potentially prognostic variables (such as site of arrest, age) to be unbiased, but largely unclear on the risk of bias in neurological outcome measurement and loss to follow-up. The assessment of confounding factors and multivariable analyses varied hugely between studies, and defied meta-analysis.

### Survival

Owing to the heterogeneity of results by subgroup (e.g. location or nature of arrest), an ‘overall’ survival estimate is clinically meaningless.

#### Location of arrest

**Fig 2:**
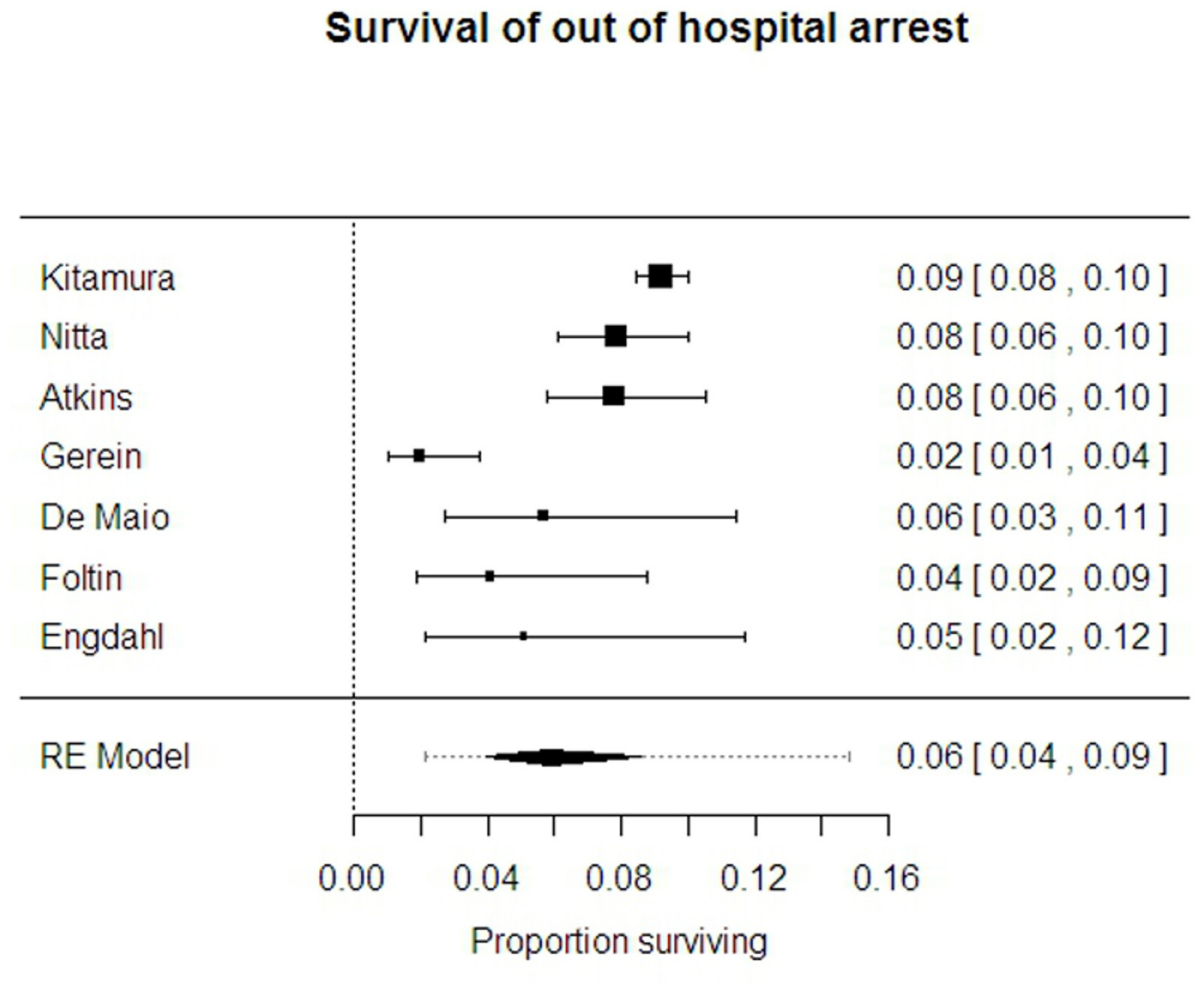
Survival from out of hospital arrest.

Survival from out of hospital arrest, largely at hospital discharge, is reported by seven studies in 7223 patients[1, 2, 10, 16, 19, 20, 26]. The overall average survival was 5.8% (95% CI 3.9% to 8.6%) but there was marked heterogeneity between the studies (95%PrI 2.1% to 14.8%, I-sq 81%). (Fig 2)

**Fig 3:**
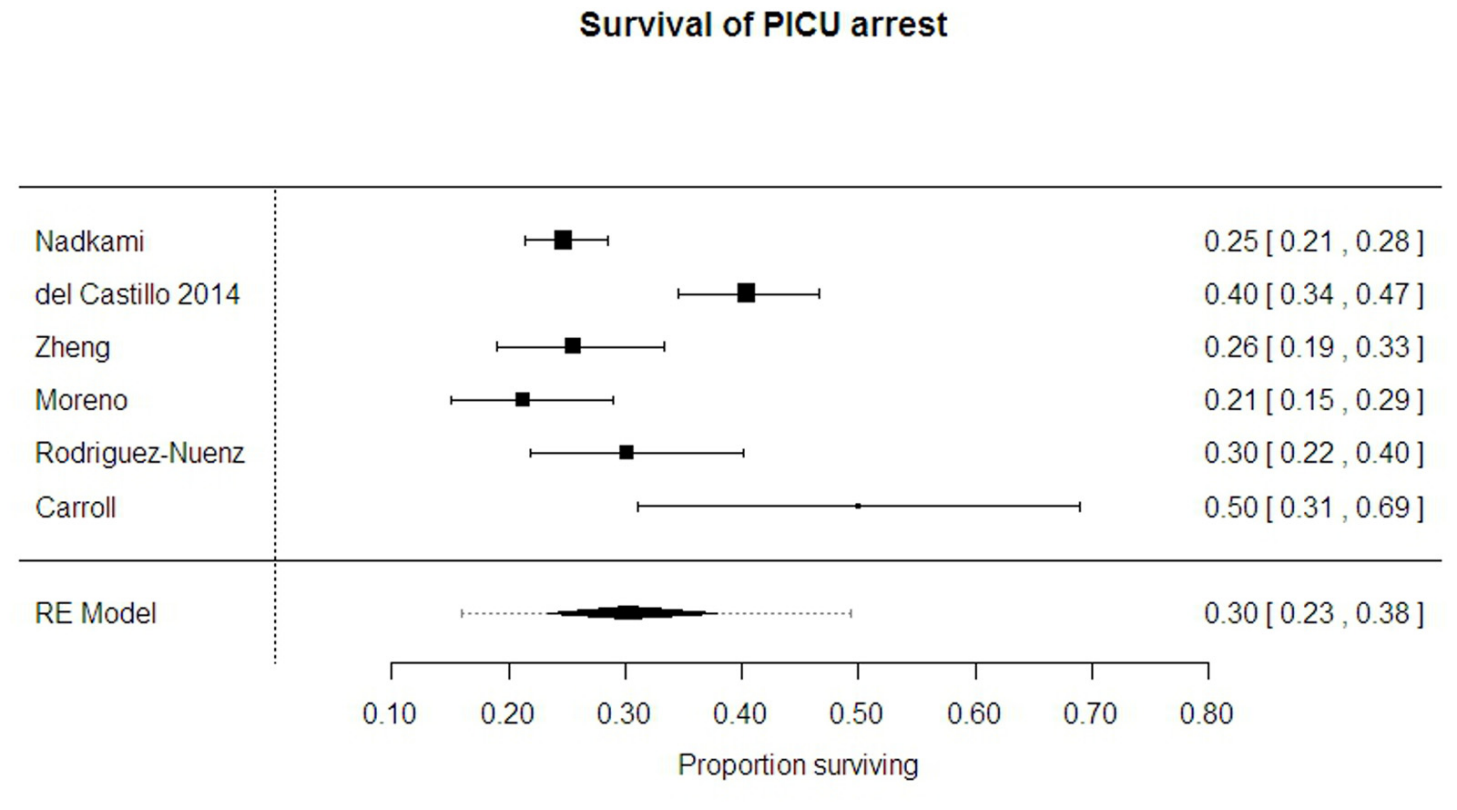
Survival from PICU arrest.

Survival from arrest in PICU carried a better prognosis; on average 30.1% survival to discharge (95% CI 23.4% to 37.9%, PrI 16.0% to 49.3%, I-sq 83%). This was reported by six studies[3, 9, 15, 18, 25, 28] over 1213 arrests. (Fig 3)

**Fig 4:**
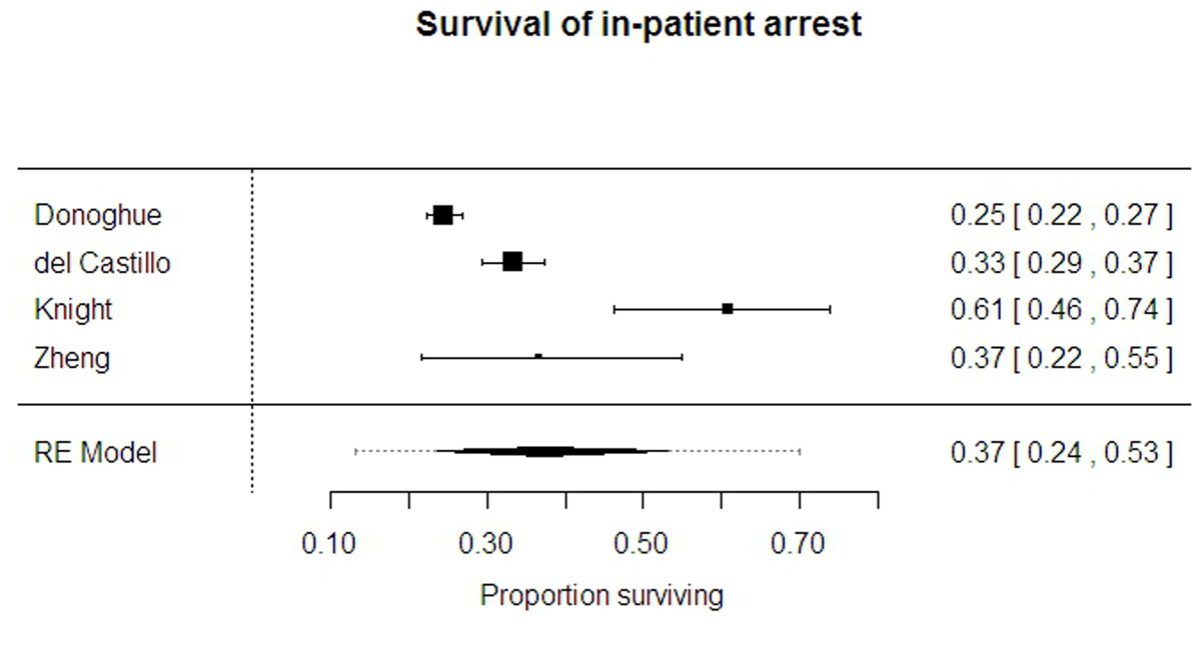
Survival from in-hospital arrest.

Four studies[3, 11, 17, 22] reported on 2108 in-patient arrests, showing very variable results; the location-mix of arrests varied with different proportions occurring within ICUs. The estimate average survival was 37.2% (95% CI 23.7 to 53.0%) but with a very broad prediction interval (95%PrI 13.1 to 69.9%, I-sq 96%). (Fig 4)

#### Age at arrest

**Fig 5:**
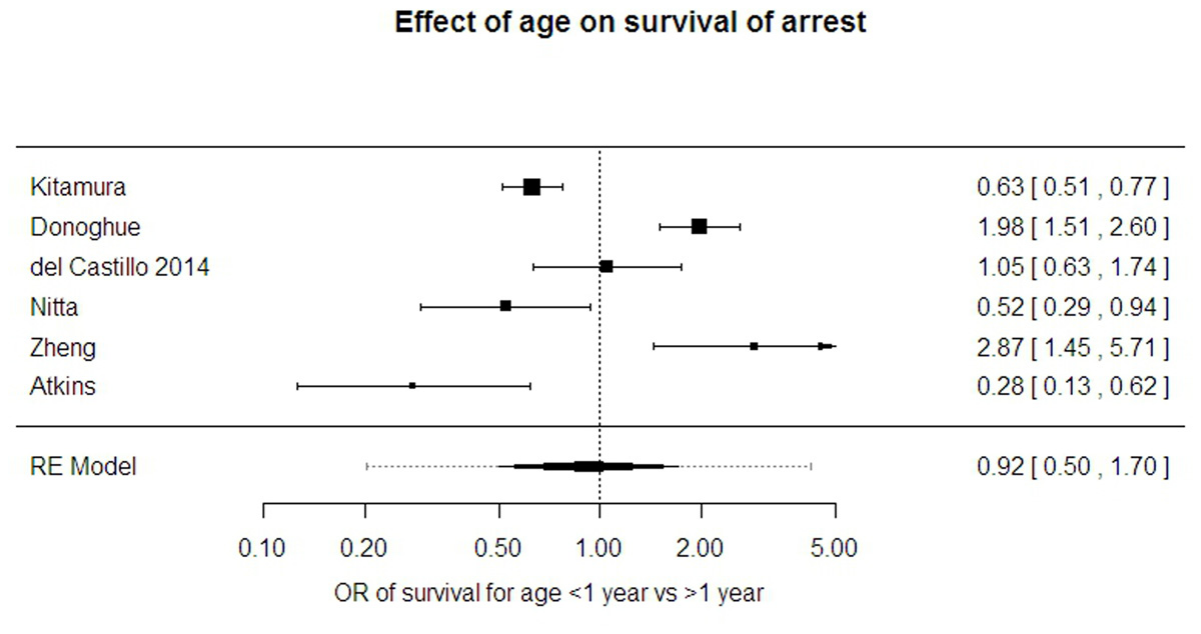
Effect of age on survival from arrest.

The effect of being under or over one year of age was assessed in six studies [1, 3, 10, 11, 18, 26] of 8010 patients. (Fig 5)

Three studies described a strongly protective effect of age >1 year, two demonstrated the opposite effect and one study described no effect of age. The estimated average OR for survival when aged <1year was 0.92 (95% CI 0.5 to 1.7) with very marked heterogeneity (95% PrI 0.20 to 4.23, I-sq 96%).

#### Nature of the presenting rhythm

**Fig 6:**
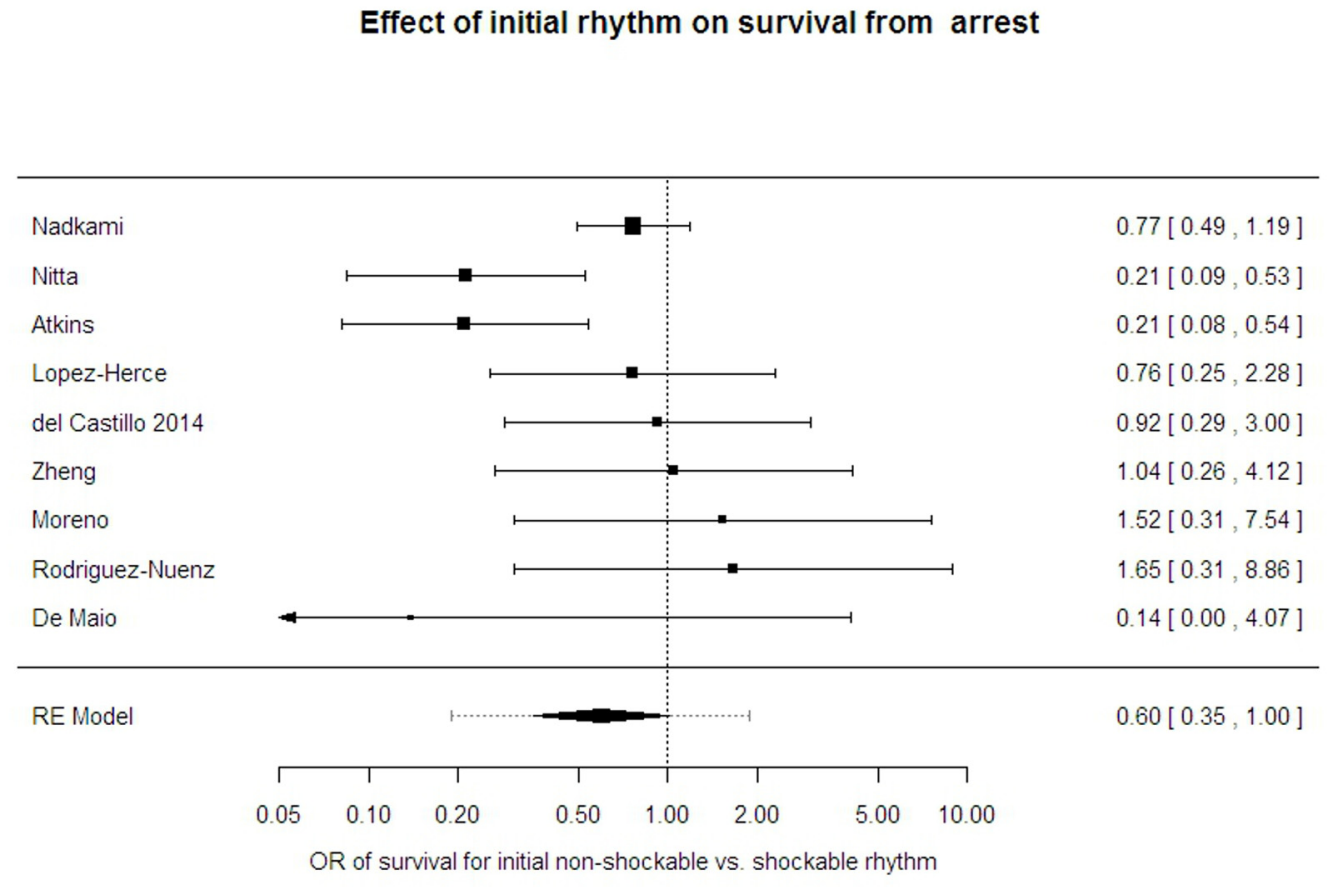
Effect of initial rhythm on survival from arrest.

Information on 2372 patients in 9 study reports[1, 3, 9, 16, 18, 24–26, 28] was available. There was variation, with the average OR 0.59 (95% CI 0.35 to 1.00) but high heterogeneity (I-sq 49%) and wide prediction intervals (95% PrI 0.19 to 1.87). One further paper undertook an analysis of the energy delivered for those with a shockable (ventricular tachycardia or fibrillation) arrest[27], but was too small to give meaningful results. (Fig 6)

#### Duration of resuscitation efforts

Three papers reported on the duration of CPR and survival rates, but used different categories of duration, precluding pooling. Each demonstrated a better outcome in those resuscitations which were shorter: Lopez-Herce[24] showed 56% mortality in resuscitations of <4 minutes rising to 100% mortality in those lasting >20 minutes, Zheng[3] showed 58% in the <10 minute group, and 95% in >30 minutes, and del Castillo[18] found 33% mortality in <5 minutes and 95% when lasting >30 minutes.

#### Neurological outcomes

Neurological outcome following cardiac arrest was detailed in 9 papers, [2, 3, 9, 10, 15, 18, 25, 26, 28]comprising 7538 patients. Quantifiable measurement of neurological status was achieved by using one of two scales (Pediatric Cerebral Performance Category, Glasgow-Pittsburgh Cerebral Performance Category). The studies were diverse and reported results differently (see Table 1).

**Table 1:** Neurological outcomes scales.

Study	Scale used	Age	Rhythm	Type of CPR	Witnessed or not	In-patient	OOH only	PICU only
Carroll	PCPC							X
Nitta	G-P	X	X				X	
Moreno	PCPC							X
Kitamura	G-P	X		X	X		X	
Nadkami	PCPC		X					
Rodriguez-Nuenz	PCPC							X
Engdhal	G-P						X	
del Castillo	PCPC							X
Zheng	PCPC					X		

PCPC = Pediatric Cerebral Performance Category, G-P = Glasgow-Pittsburgh Cerebral Performance Category, OOH = out of hospital, PICU = paediatric intensive care unit

For the purposes of comparison, “good” neurological outcome, as defined by most of the studies, was either Normal functioning, or Mild or Moderate disability. Those who were brain dead, in coma/vegetative state or with severe disability (defined as being dependent on others for daily support due to impaired brain function) were “not good” outcome. For studies reporting the individual cerebral performance scores similar results could be ascertained by dichotomising. Two of the papers[15, 26] documented cerebral performance prior to arrest, and included patients as “good” outcome if their CPC was unchanged even if they were worse than the “moderate disability” category.

#### Location of arrest

**Fig 7:**
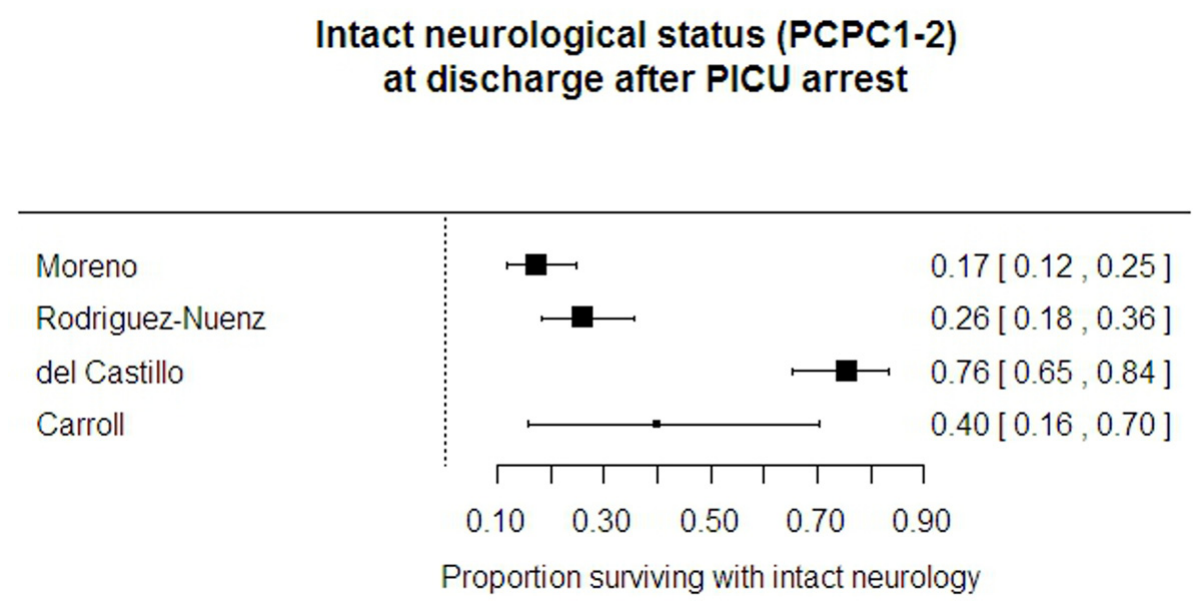
Intact neurological status after discharge from PICU arrest.

Out of hospital survival with “good” neurological outcome was 3%, similar across all three studies that reported it (2.8% according to Nitta[26], Kitamura[10] states 3.2% and Engdahl[2] reports 3.1%). Data reported for PICU patients reported on 324 patients included in four papers.[15, 18, 25, 28] Their reports of good neurological outcome were very varied, between 17% and 71%, though the value of 71% in del Castillo[18] was missing information on 15/101 (15%) of patients. (Fig 7)

#### Age at arrest

Two studies assessed the effect of age; >1 year old was associated with an increased chance of a “good” cerebral performance category outcome (Nitta[26] OR 3.79 (95% CI 1.26 to11.38) Kitamura's [10]adjusted OR 1.60 (1.07–2.36))

#### Nature of the presenting rhythm

Two studies categorised patients by presenting rhythm; the numbers with shockable rhythm were small, and their results very different. One study suggested a huge advantage to presentation with a shockable rhythm: (Nitta[26] adjusted OR 14.51 (95% CI 11.09 to19.00)), the other a marginal, and non-significant, advantage (Nadkami[9] OR 1.38 (95% CI 0.82 to2.31)).

#### Duration of resuscitation efforts

No study detailed duration of arrest efforts and neurological outcome.

#### Other features

One study reported the type of CPR (if any) given by the bystander at witnessed arrests. Any CPR had a better neurological outcome compared to no CPR[10], adjusted OR 2.59 (95% CI 1.81 to3.71) and conventional CPR (with ‘mout to mouth’ ventilation) had a better neurological outcome compared to compression only CPR for patients with a non-cardiological cause of arrest (OR 2.22 (95% CI 1.40 to 3.57). The same data set from Japan showed reduced survival rates if the arrest occurred at night compared to day (adjusted OR 0.68; 95% CI: 0.56 to 0.82), or if arrests occurred on weekends/holidays compared to normal working week days[29] (adjusted OR 0.79; 95% CI, 0.65 to 0.97), and improved outcomes if the telephone contact provided instructions to the on-the-scene witness of the arrest[21].

## Discussion

This systematic review (with meta-analysis of relevant studies) reports on the outcomes of paediatric cardiopulmonary arrest from published data from more than 18,000 episodes, in 16 data sets. However, even with such a sizeable amount of information, there is a surprising lack of data that can be used to look for predictors of successful cardiopulmonary outcomes, owing to the heterogeneity of the various study populations and the different outcome measures that are reported. Meta-analysis was only possible for survival outcomes, and one neurological outcome, as others were reported too inconsistently for meaningful statistical pooling. Many of the studies reported only short time outcomes such as discharge from PICU or from hospital, there being fewer papers that have looked at long term outcomes, e.g. neurological status at six months or at a year post-event.

The review supports that in-hospital patients, in particular those on an intensive care unit, have a better chance of survival following cardiopulmonary arrest, and probably better neurological outcome than those cases of out-of-hospital arrest. Interpreting the neurological outcome data in these groups is difficult, partly through selective outcome reporting. This phenomenon can be thought of as a type of within-study ‘publication bias’; the classic understanding of publication bias is that studies with unfavourable results will not be published, over-estimating the effects of an intervention. In this instance, as two of four studies reporting age effects revealed improved neurological outcome, the other studies did not report this measure, but it is unclear if this had been examined in these whether the same effect would have been seen.

Individual studies have assessed how variation in quality of resuscitation can affect outcome, by presuming epidemiological variation in training of provision in- and out-of normal working hours[29], or by telephone instruction of CPR[21] or by group training and adherence to guideline policies[22, 27]. These differences may change the absolute numbers surviving with improved neurological status, there is little to suggest that they alter the relative predictive value of other factors.

Greater attention to patient focussed outcome measures is indicated in future work to assist in the decision making by clinicians and parents/carers alike. Any advance on understanding which elements are most predictive and potentially modifiable must come from analysis of large, pooled, individual patient data with unified and clearly defined outcomes measures.

There were few data to help answer our primary question of; at what point are resuscitation attempts futile and should be discontinued? More research will be required about the duration on resuscitation outcomes and answers may become available by the use of national registries. By looking at these registries, in parallel and in combination in long term studies, reliable and accurate predictors could potentially be determined that could help in the managing of a child with cardiopulmonary resuscitation.

There is a need to collaboratively, prospectively, collect potentially predictive data on these rare events and make good use of opportunities to discover patient-relevant long-term outcomes. This may be possible by harnessing the systems which already capture information on patients who suffer cardiac arrest, and their onward health care journey through routine clinical record or billing systems. Such data should be treated with care, and analyses undertaken with the guidance of parent/patient groups to highlight which are the most meaningful outcomes. Before trying to use this information in the critically stressful environment of an arrest and attempt at resuscitation, compassionate and academically robust studies are required to examine how such information may be understood and considered in making a shared decision about continuing with active care.

## Appendix 1: Example search strategy for Medline

1. Heart Arrest/ or Resuscitation/ or Cardiopulmonary Resuscitation/ or Heart Massage/ or Out-of-Hospital Cardiac Arrest/
2. Advanced Cardiac Life Support/ or Life Support Care
3. compression/ or cardiopulmonary insufficiency/ or heart massage/ or artificial ventilation/
4. CPR.mp. [mp = ti, ab, tx, kw, ct, ot, sh, hw, tn, dm, mf, dv, nm, kf, ps, rs, ui]
5. Resus*.mp.
6. (Chest adj2 Compression*).mp.
7. (Cardi* adj2 arrest*).mp.
8. (respirat* adj2 arrest*).mp
9. (Basic adj2 Life Support).mp
10. (Paediatric adj2 Life Support).mp.
11. (PLS or BPLS or BLS).mp
12. (Advanced adj2 Life Support).mp
13. (APLS or PALS or ALS or PILS or ILS).mp
14. 1 or 2 or 3 or 4 or 5 or 6 or 7 or 8 or 9 or 10 or 11 or 12 or 13 or 14
15. (Infan* or newborn* or new-born* or perinat* or neonat* or baby or baby* or babies or toddler* or minors or minors* or boy or boys or boyfriend or boyhood or girl* or kid or kids or child or child* or children* or schoolchild* or schoolchild or adolescen* or juvenil* or youth* or teen* or under*age* or pubescen* or pediatric* or paediatric* or peadiatric* or prematur* or preterm*).mp. [mp = ti, ab, tx, kw, ct, ot, sh, hw, tn, dm, mf, dv, nm, kf, ps, rs, ui]
16. school child.ti. or school child.ab. or school child*.ti. or school child*.ab. or school.ti. or school.ab. or school*.ti. or school*.ab.
17. Pediatrics/
18. 15 or 16 or 17
19. Prospective Studies/ and Cohort Studies/
20. Prospective.mp.
21. Cohort.mp.
22. 20 and 21
23. (Prospective* adj2 Cohort*).mp. [mp = ti, ab, tx, kw, ct, ot, sh, hw, tn, dm, mf, dv, nm, kf, ps, rs, ui]
24. 19 or 22 or 23
25. 14 and 18 and 24
26. limit 25 to yr = "2003-Current"

## Supporting Information

S1 File PRISMA 2009 Checklist. (DOC)

S1 Table Extracted mortality data. (CSV)

S2 Table Extracted neurological morbidity data. (CSV)
